# Hepatic Colic Cured by Chloroform

**Published:** 1875-11

**Authors:** Q. C. Smith


### HEPATIC COLIC CURED BY CHLOROFORM.

BY Q. C. SMITH, M. D.

In order to give the "pith and point" of this case as briefly as possible, we will merely present the outlines, leaving the dates and other minutiæ to be filled in by the imagination of the reader.

About seven months since, we were called to treat the case of a gentleman about sixty years of age, who had been affected about twenty-five years with pain, enlargement and induration of the liver.

He had, at times, more or less frequently, suffered from paroxysms of hepatic colic. During the last few years his hepatic troubles had rendered his life miserable most of the time.

We found him suffering a severe paroxysm of hepatic colic; skin and eyes intensely jaundiced, urine dark and smoky-looking, liver greatly enlarged, tender and indurated. We ordered twenty grains of ipecac, and half-tablespoonful doses of chloroform every hour, until the paroxysm should be relieved. Two doses of the chloroform and one of the ipecac (which did not cause emesis) were sufficient. We then instituted such treatment, constitutional and otherwise, as is recommended by standard authorities—Flint, Watson, Trousseau, etc.—and ordered the chloroform to be given as before, should another paroxysm occur. Despite our efforts, the paroxysms continued to recur, more or less frequently, for about three weeks, generally from one to three days apart, and often so severely as to cause syncope.

Beginning to think the case incurable, and believing it our duty to let him die as easy as possible, if die he must, as a dernier resort, we resolved to try the anodyne and antispasmodic powers of chloroform to their full extent.

We ordered half a tablespoonful of chloroform, in a little water, every two hours, until pain was entirely relieved, and then continued it as often as necessary to keep him stupefied. We saw him daily, and continued the chloroform treatment, and also moved the bowels freely, with large doses of elaterium, (nothing milder would move them,) for three days and nights; then gradually ceased to give the chloroform, and allowed the patient to regain his natural status. We had watched carefully, from the beginning, for biliary calculi, but found none until the last alvine evacuations, during the chloroform stupefaction, when a large number were passed, but none larger than a common garden-pea. No more paroxysms occurred.

Our patient was now prostrated to an extreme degree—unable to speak above a whisper, and only a few words at a time. Pulse sixty to seventy; temperature normal. During the chloroform stupefaction the urine was as clear as spring-water—the nervous urine of pathologists.

We now ordered a mild, pleasant tonic of muriatic acid and bark, and left off the use of all other medicines, with directions to resume the chloroform should the paroxysms show a disposition to return.

Under the tonic treatment, he slowly improved; the secretions and excretions became normal and regular; the skin cleared up and the appetite returned. The bowels moved regularly without a purgative, a condition that had not existed for years before, and the alvine evacuations were of good appearance. He continued slowly but steadily to improve, under the aforementioned tonic treatment, for about six weeks; was then able to walk out in the yard, and ride short trips in a carriage. At this juncture, a looked-for and dreaded complication appeared—dropsical effusions in the extremities. The patient's legs and arms swelled from anasarca up to his body, and were quite painful, especially during the day, when he moved about.

We continued the tonic treatment; ordered him to scrub his whole person with soap, and cool water fresh from the fountain, twice a week, paint the extremities with iodine daily, and drink as much strong decoction of elder-root bark as his stomach would bear. Under this treatment, the dropsical effusion and hepatic troubles entirely disappeared in a few weeks; and now, despite the advanced age of my patient, he cuts, saws and splits wood daily in the forest, and declares he is a sounder man than he has been for twenty-five years.

Let us accord merit when merit is due, and score one for chloroform, while so many are being scored against it.—*Pacific Medical and Surgical Journal.*

